# An N‐of‐1 study of daily alcohol consumption following minimum unit pricing implementation in Scotland

**DOI:** 10.1111/add.15382

**Published:** 2021-01-12

**Authors:** Dominika Kwasnicka, Massoud Boroujerdi, Aileen O'Gorman, Martin Anderson, Peter Craig, Louise Bowman, Mark McCann

**Affiliations:** ^1^ Faculty of Psychology SWPS University of Social Sciences and Humanities Wroclaw Poland; ^2^ NHMRC CRE in Digital Technology to Transform Chronic Disease Outcomes Melbourne School of Population and Global Health, University of Melbourne Melbourne Australia; ^3^ MRC/CSO Social and Public Health Sciences Unit University of Glasgow Glasgow Scotland, UK; ^4^ University of the West of Scotland Paisley Scotland, UK; ^5^ Scottish Drugs Forum Glasgow Scotland, UK

**Keywords:** Alcohol, alcohol policy, behaviour change, ecological momentary assessment, minimum unit price, N‐of‐1

## Abstract

**Background and aims:**

Within the context of Scotland's policy change to implement a minimum unit price (MUP) per unit of alcohol sold in licensed premises, this study used an N‐of‐1 design to assess between‐person differences in the psychological and social factors associated with daily alcohol consumption.

**Design and setting:**

A mixed‐methods approach combined N‐of‐1 observational studies, comprising daily surveys followed by qualitative social network interviews (not reported here). Peer researchers with lived experience of substance use were involved in the study design and fieldwork was conducted in towns and rural areas in the East of Scotland.

**Participants/cases:**

Twenty‐five adults with current or recent history of alcohol dependence recruited for three 12‐week waves: 11 in wave 1 (pre‐MUP), 11 in wave 2 (pre‐ and post‐MUP) and three in wave 3 (post MUP).

**Measurements:**

Gender, age, alcohol and other drug use history. Daily surveys for 12 weeks captured information about factors in the last 24 hours, e.g. amount and type of alcohol consumed, stress, social contact.

**Findings:**

Each participant was in the daily survey for a mean of 64 days [standard deviation (SD) = 42; median = 59], with a response rate of 48%; 15 participants provided sufficient data for analysis. Factors related to daily alcohol consumption differed between individuals. Models suggested that some individuals with high initial consumption reduced drinking after MUP, but explanatory factors differed, e.g. changing motivation was important for some, while alcohol availability was important for others.

**Conclusions:**

Adapting N‐of‐1 methods for an observational study uncovered differences in alcohol consumption change before and after minimum unit pricing implementation in Scotland, evidence of individual differences in the factors relating to alcohol consumption patterns and some evidence that post‐MUP consumption changes may be related to changing psychosocial factors.

## Introduction

The Scottish Government implemented legislation for Minimum Unit Pricing (MUP) so that off‐sales alcohol cannot be sold to the public below 50 pence per unit of alcohol. The law came into effect on 1 May 2018. MUP aims to increase the price of the cheapest alcohol, which is theorized to lower consumption among the heaviest drinkers and thus reduce alcohol harms [1]. Other studies evaluated MUP by looking at population level trends in price, consumption and theorized change processes [2]. This study used an N‐of‐1 design to understand the individual‐level factors which in aggregate lead to population level trends.

The N‐of‐1 design facilitates ‘precision’ behaviour change studies examining within‐person variability over time by repeatedly measuring a set of factors within the same individual [3]. N‐of‐1 contrasts with traditional research methods that aim to ascertain between group differences rather than within‐person variability [4]. At its core, the N‐of‐1 approach is a case study of the factors that explain trajectories of change in one individual. Rather than searching for regularities averaged over a population, an N‐of‐1 design, run in parallel across several individuals, gives information about the nature and extent of differences between individuals. Such an approach can provide additional information about the breadth of plausible causal mechanisms that underpin a theory of change [5]. From a systems perspective [6], it can give insight into the diversity of agent behaviours—and their dynamics—that may underpin system change and that interact with system structure. In this study, we have implemented the N‐of‐1 as an observational design looking at individual change mechanisms within the context of a change in the policy environment [7].

This N‐of‐1 study can help to more clearly theorize individual change processes around consumption. The study asked two questions: (1) what are the individual and social determinants of within‐person change in alcohol use; and (2) what contextual and environmental factors are related to alcohol consumption, particularly MUP implementation? 1

## Methods

### Design

We used a mixed‐methods approach to collect information for each N‐of‐1 case. This included smartphone‐administered daily ecological momentary assessment (EMA) surveys [8], followed by a qualitative interview with an egonet social network component [9]. Egonet and qualitative data analyses will be presented in a separate paper. We adopted a participatory approach to the study [10]: working with a team of peer researchers with lived experience of substance use to develop questions, recruit participants and conduct the fieldwork. We used a Delphi workshop and peer feedback to develop EMA questions and study materials (see Supporting information, Appendix S1).

### Participants

Inclusion criteria were adults who self‐identified as ‘heavy drinkers’, i.e. drinking to a level that is likely to harm their health, or former heavy drinkers who identified as being ‘in recovery’, abstinent or reducing their alcohol use. Exclusion criteria were being unable to provide informed consent and literacy or language barriers preventing participation.

### Procedure

The Scottish Drugs Forum (SDF) peer research team recruited participants to the study, administered the study materials, obtained consent, explained how to operate the smartphone and complete the daily survey, maintained contact throughout the study, safeguarded and signposted and conducted follow‐up interviews. Participants were recruited via the peer team's contacts within their local communities. Confidentiality and anonymity were maintained for the participants. No identifying data on the participants were included in the survey or interview data submitted for analysis. Survey data were anonymized using a number to indicate the survey wave in which they were collected (e.g. 1007, 2005).

Those participating completed a baseline survey including information on gender, age, alcohol and other drug use history. They received daily surveys to their mobile phone or to a study phone for 12 weeks, sent at 7 p.m. daily. There were three waves of 12‐week survey periods. The first wave was a 12‐week period before MUP (1 February), the second started before (1 April) and finished after MUP, and the third started after MUP was implemented (1 June). Participants received the same survey each day (Supporting information, Table S2) with question ordering randomized, and an open text question at the end of each survey to add other information relevant to drinking or general wellbeing. The questions asked about the previous 24 hours, e.g. amount and type of alcohol consumed, stress and social contact. After 12 weeks of completing the EMA study, participants were invited for an interview to talk about their experiences during the survey. The study took place in rural areas and intermediate‐sizes towns in the East of Scotland. Anonymized data are available via the Open Science Foundation (osf.io/ESW4D).

### Data analysis

For the EMA data analysis, missing values were imputed for the data from respondents with moderate (50%) or high (60%) quality data using R's Amelia II [11] package. This approach is equivalent to multiple imputation by chained equations as applied to more common data sets. However, rather than using prediction equations based on other participants’ data, the predictions are based on each individuals’ own data provided on other days of the study. For example, if a participant's rating of their mood was missing for 1 day, their mood would be estimated based on their rating on the previous day and the following day, conditional upon other factors such as stress or temptation. An important distinction to traditional imputation is that we exclude other participants’ data when estimating these predictions; this removes the assumption any individual has similar patterns in their data to other participants, and missing data do not trend towards the sample average. Multi‐level models and correlations are based on 50 imputed data sets; plots are based on the first imputed data‐set. We produced plots of the unimputed responses over calendar time for each variable for each respondent to show the temporal trends in responses (see Supporting information, Table S2).

### Individual participant analysis

For individual participant analysis, the analytical data set comprised linear regression residuals of the current day's variable value, conditioning on the previous day's value (‘1‐day lag’ residuals) [12]. We considered, but ruled out, a 7‐day lag as preliminary analysis did not identify a weekend drinking seasonal trend. We calculated the partial correlation of each variable with alcohol units, conditioning on the other variables using R's ‘miceadds’ package [13]. The variables used were mood, motivation, temptation, effort, stress, situations when alcohol is available and total units of alcohol.

### Association of MUP with alcohol consumption

**Figure 1:**
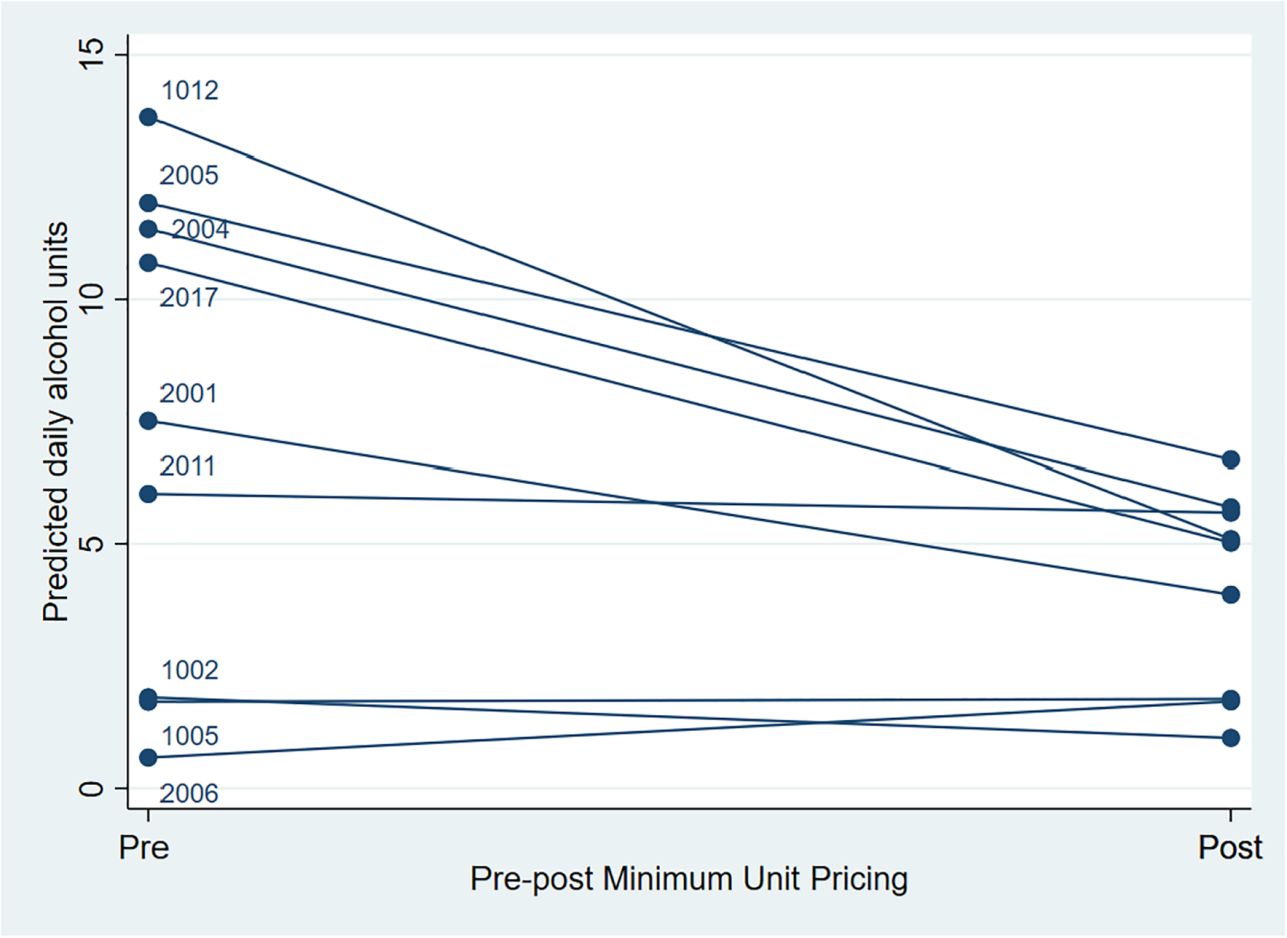
Individual differences in pre–post minimum unit price (MUP) alcohol consumption for nine respondents who participated in pre–post study waves and provided sufficient data [Colour figure can be viewed at wileyonlinelibrary.com]

For the analysis of the association of MUP with consumption, we fitted multi‐level models (MLMs) to the longitudinal data [14]. Observations on each day were considered level 1 and respondent at level 2. A binary variable indicating the introduction of MUP was added to the independent variables; the coefficient of this variable shows the effect of MUP on daily consumption. The analysis focuses upon the variables that were fully reported in the data set; variables that had incomplete information (the influence of money on substance use and social contact with other people) were excluded from further analysis. In line with the N‐of‐1 approach, this pooled analysis aims to describe the associations between variables among the study respondents, rather than drawing inference about associations in the wider population. The MLM equation is:

$$\text{Total}\;{\text{alcohol units}}_{\mathrm{ij}}=\left(\beta_{0}+u_{\text{0i}}\right)+\left(\beta_{1}+u_{\text{1i}}\right)\;{\text{time}}_{\mathrm{ij}}+\beta_{2}x_{\mathrm{ij}}\ldots ,$$

where *i* is the subject and *j* is the occasion (time). The *u*_*i*_ is the random variance accounting for differences in individual estimate for β_1_. β_2_ is the coefficient indicating post‐MUP implementation. We also tested for a random slope for between‐person variation in the effect of MUP, incorporating a slope variance and intercept–slope covariance parameters. The model is based on 15 participants. As we cannot draw a before‐and‐after trend line for the six participants who did not provide pre–post data, the plot in Fig. 1 is based on the nine participants providing before‐and‐after data (model reported in Supporting information, Table S5). We found no evidence of switching to higher use of other drugs, and no evidence of greater contact with services after MUP (Supporting information, Table S3).

### Factors related to MUP change

We conducted subsequent exploratory analysis to explore how the changing MUP context related to individual determinants of consumption. Using the individual participant data sets, we fitted a series of regression models including an MUP coefficient showing the standard deviation (SD) change in consumption before and after MUP, and how this coefficient changed after including factors predictive of each individual's drinking identified from the partial correlation analysis. The paper presents this analysis for the participants whose consumption fell after MUP; full results are in the Supporting information. These analyses were not pre‐registered and the results should be considered exploratory.

### Ethics approval

Ethics approval was granted on 13 January 2018 by the University of Glasgow Medicine Veterinary and Life Sciences Ethics Committee, ref. 200 170 077.

## Results

In total, 25 respondents took part in the study across three 12‐week waves; 11 in the 1st wave (pre‐implementation, and two participants chose to continue for longer than 12 weeks), 11 in the 2nd wave (pre‐ and post‐implementation) and three in the 3rd wave (post‐implementation). On average, each participant was in the EMA study for 64 days (SD = 42; median = 59); and on average each participant provided 27 responses (SD = 26; median = 21); the total response rate for the full participating sample was 48% (SD = 23; median = 51). For six respondents, alcohol was their only substance of dependent use, while six reported secondary dependence on substances other than alcohol. Most respondents (68%) lived alone, two were homeless and five reported having had no social contact in the previous six weeks (see Supporting information, Table S3).

### Missing data

In total, the study respondents were in the EMA study for 1514 days when they could provide measures. Days in the study and relative response rate varied between individuals. After excluding those with high missing data, 15 respondents were deemed suitable for further quantitative analysis and missing data was imputed separately for each respondent's data.

### Individual participant analysis

Table 1 shows the partial correlations for each variable's association with daily consumption, and the mutually adjusted linear regressions for the same data. For clarity, we removed coefficients with little evidence for a robust association (*P* > 0.05).

**Table 1:** Partial correlations (top) and mutually adjusted standardized regression coefficients (bottom) for individual participant data in relation to daily alcohol units consumed

Correlations	1002	1004	1005	1006	1007	1009	1012	2001	2004	2005	2006	2011	2017	3003	3006
Mood			−0.18		−0.45	−0.35		0.47			−0.24				
Motivation	−0.31	−0.48	−0.46		−0.26	−0.53	−0.26	−0.25			−0.59				−0.47
Tempted	0.17	0.39	0.24		0.41	0.48				0.33	0.26		0.34	0.69	0.25
Effort	−0.88	−0.88	−0.27			−0.41		−0.36		−0.51	−0.89			−0.44	−0.57
Stress			0.21		0.37										
Alcohol situations			0.41	0.25	0.31	0.72	0.76		0.48	0.65		0.88		0.71	0.44

Regression coefficients	**1002**	**1004**	**1005**	**1006**	**1007**	**1009**	**1012**	**2001**	**2004**	**2005**	**2006**	**2011**	**2017**	**3003**	**3006**
Mood			−0.16					0.46							
Motivation			−0.37					−0.25							
Tempted														0.52	
Effort	−0.83	−0.9						−0.22		−0.37	−0.84				−0.39
Stress												−0.17			
Alcohol situations			0.38		0.28	0.6	0.76		0.46	0.52		0.9		0.44	0.33

Coefficients presented where estimate *P*‐value < 0.05.

There were substantial differences between individuals in terms of the factors that were associated with their alcohol consumption; for participant 1005 all the variables were correlated with consumption, and three remained associated in the mutually adjusted model. For 1006 and 2017, only a single variable was correlated with consumption. The direction of associations was consistent for all respondents with the exception of mood, which was positively correlated with drinking for 2001 and negatively correlated for other respondents. Situational availability and effort to reduce drinking were the factors most commonly associated with consumption among respondents.

### Association of MUP with alcohol consumption

Multi‐level models were fitted for 15 respondents. The changes over time in consumed total units of alcohol (the outcome) with stress, tempted, motivated, effort, mood, drink‐feeling (whether they perceived their drinking positively or negatively) and number of contacts as independent variables were analysed with MLM (Table 2).

**Table 2:** Multi‐level models for combined sample data with alcohol units consumed as the outcome for 15 respondents

Parameter	Model 1	Model 2	Model 3	Model 4	Model 5	Model 6	Model 7	Model 8
Time	−0.01 (−0.02, 0.01)	−0.01 (−0.02, 0.01)	0 (−0.02, 0.01)	0 (−0.02, 0.01)	−0.01 (−0.02, 0.01)	−0.01 (−0.03, 0.01)	0.00 (−0.02, 0.01)	0.00 (−0.01, 0.01)
Post‐MUP	−1.81 (−3.29, −0.33)	−1.83 (−3.3, −0.37)	−1.69 (−3.1, −0.27)	−1.9 (−3.31, −0.49)	−1.54 (−2.94, −0.15)	−1.67 (−3.14, −0.20)	−0.53 (−1.81, 0.76)	−0.56 (−1.8, 0.69)
Mood		−0.03 (−0.05, −0.01)						0 (−0.02, 0.02)
Motivation			−0.09 (−0.11, −0.07)					−0.03 (−0.06, −0.01)
Tempted				0.07 (0.05, 0.08)				0.02 (0, 0.04)
Effort					−0.11 (−0.13, −0.09)			−0.07 (−0.1, −0.05)
Stress						0.04 (0.01, 0.06)		0.02 (−0.01, 0.04)
Alcohol situations							0.12 (0.11, 0.14)	0.12 (0.1, 0.13)
Intercept	5.71 (3.75, 7.66)	7.12 (4.97, 9.26)	11.47 (9.43, 13.51)	1.71 (−0.05, 3.48)	12.75 (10.69, 14.82)	3.5 (1.25, 5.75)	1.87 (0.37, 3.38)	6.8 (3.74, 9.87)
Level 2 variance	12.74	11.05	7.58	5.59	6.1	10.79	6.62	1.04
Level 1 variance	60.67	60.4	56.85	58.74	55.44	60.18	47.15	0.86
ICC	0.17	0.15	0.12	0.09	0.1	0.15	0.12	0.55

MUP = minimum unit price; ICC = intracluster correlation coefficient.

Before adjusting for other factors, daily alcohol units after MUP reduced by 1.80 on average, but with no evidence of a downward trend for the sample as a whole [95% confidence interval (CI) = –3.29, 0.33]. When analysed collectively for the sample, mood, motivation and effort were all associated with lower daily consumption, and temptation, stress and—most strongly—situational availability of alcohol were associated with higher consumption. Approximately 15% of the variation in daily consumption was between‐respondent variation, suggesting between‐individual differences (some people drinking a great deal continually, others drinking very little) were quite high, although with substantial day‐to‐day variation for each individual. Figure 1 shows the extent of between‐person variation in post‐MUP change in consumption for the nine participants providing data before and after MUP was implemented. Four participants showed a reduction, two participants showed stable consumption before and after MUP and three remained at zero or very low consumption before and after MUP.

### Factors related to MUP change

For the five participants reporting a reduction in alcohol, the unadjusted coefficient ranged from a 0.3 to 0.98 SD reduction in daily alcohol units after MUP (Table 3). The extent to which psychosocial factors explained the drop in consumption differed for each participant. For 2017 participants who reported the greatest reduction in consumption post‐MUP (−0.98), the sole psychosocial predictor of consumption was temptation. After its inclusion in the model, the MUP coefficient reduced by 14%. Change in temptation could only account for a small proportion of consumption change. For 2005, participants who had the lowest MUP change among those with a downward trend (−0.32), including the predictors tempted, effort and alcohol situations entirely nullified and, in fact, reversed the direction of the MUP coefficient. This pattern appeared for those who had no trend towards reduced consumption and may be due to the additional variables introducing noise into the estimation of a null coefficient where there is no evidence of an underlying MUP effect.

**Table 3:** Regression coefficients of individual participant data showing the standard deviation change in daily units after MUP and the change in the post‐MUP coefficient after accounting for psychosocial factors

	1012			2001			2004			2005			2017		
	Coef	*P*‐value	Coeff. change	Coeff.	*P*‐value	Coeff. change	Coeff.	*P*‐value	Coeff. change	Coeff.	*P*‐value	Coeff. change	Coeff.	*P*‐value	Coeff. change
Model															
Unadjusted	−0.751	0.011	0	−0.464	0.04	0	−0.582	0.01	0	−0.321	0.258	0	−0.981	0.001	0
+ Mood	–	–	–	−0.261	0.215	0.44	–	–	–	–	–	–	–	–	–
+ Motivation	−0.513	0.145	0.32	−0.215	0.292	0.54	–	–	–	–	–	–	–	–	–
+ Tempted	–	–	–	–	–	–	–	–	–	−0.149	0.604	0.54	−0.842	0.008	0.14
+ Effort	–	–	–	−0.155	0.441	0.67	–	–	–	0.003	0.99	1.01	–	–	–
+ Stress	–	–	–	–	–	–	–	–	–	–	–	–	–	–	–
+ Alcohol situations	−0.275	0.173	0.63	–	–	–	−0.178	0.427	0.69	0.24	0.282	1.75	–	–	–

Data presented for five participants with greatest change in consumption after minimum unit price (MUP) in Fig. 2. Other participant results appear in the Supporting information Appendix S1.

For the other three respondents (2001, −0.46, *P* = 0.04; 2004–0.58, *P* = 0.01; 1012 (−0.75, *P* = 0.01), their individual level predictors led to between a 63 and 69% reduction in the MUP coefficient, although the predictors themselves differed. Alcohol availability were key factors for 1012 and 2004, and its inclusion led to a large reduction, while for 2001 the MUP coefficient saw a comparable proportional reduction, but this was due to the inclusion of parameters for mood, motivation and effort.

## Discussion

The innovative use of N‐of‐1 methods in this study found differences in alcohol consumption change before and after MUP, evidence of individual differences in the factors relating to alcohol consumption patterns and some evidence that post‐MUP consumption changes may be related to changing psychosocial factors. The observed individual patterns lend some support to the theory of change for the MUP policy, but also uncovered substantial differences in how individuals may respond. While other studies can assess the effect of MUP at the national level by studying population level change in consumption and harms, this study contributes to understanding how the factors that explain consumption among the harmful drinking population differ from person to person.

We observed a small decrease in alcohol consumption after MUP implementation among the heaviest drinkers in the study, but less change among less frequent or mainly abstinent drinkers. None of the abstaining respondents experienced prolonged drinking relapses, so we cannot assess if MUP influenced maintained abstinence or controlled use. The within‐person predictors of consumption varied, with effort and situational availability appearing most commonly.

The theories of behaviour change and domain expertise that informed the study questions [15] would suggest that all the factors under study should predict drinking, yet there appeared to be differences between individuals in the factors that related to their drinking. Some of the factors suggest the importance of implementing positive coping strategies (e.g. motivation or higher effort), while for others the external environment (e.g. stress or availability) appeared more relevant.

A key assumption of the theory of change for MUP is that an increase in price will lead to lower consumption and a decline in subsequent reduced harms at the level of the whole population of Scotland. Decisions concerning continuing the MUP policy will focus upon these population level changes. N‐of‐1 analysis provides insights into what changes may occur among individuals within the population, and thus the variety of changes that underpin overall population change. We found that accounting for change in situations where alcohol was available helped to explain a proportion of reduced consumption post‐MUP for some individuals; this may reflect the change in alcohol use among their heavy drinking peers. For others, changes in psychological factors such as mood, motivation or effort explained the reduced consumption, suggesting that the awareness of the change in alcohol policy may have shifted motivations and subsequent behaviour.

It is important to emphasize that our analysis found no evidence that variables relating to perceived importance of price in drinking behaviour were associated with consumption, before or after MUP implementation. Public debate and visible change in the range of alcohol available on shop shelves around the time of MUP implementation may have changed motivations and behaviour for some heavy drinkers. However, the change process that relates reduced alcohol affordability to lower consumption does not rely upon individuals considering the importance of price and making a conscious change in purchasing decisions. Affordability, as a change in the risk macro‐environment, does not require individuals to cognitively engage with price in order to have its effect, although our analysis suggests that MUP may have led to additional individual and micro‐environmental changes in some cases. The findings from the N‐of‐1 analyses can help to inform the wider evaluation of population‐level change in consumption by helping to theorize the microlevel change processes, and help to contextualize the extent of variation in response to MUP. For example, helping to theorize various mechanisms of change among population subgroups that sit underneath the population level theory of change. They can also inform next steps and long‐term discussions around alcohol policy and treatment provision.

The mechanisms through which MUP leads to reduced drinking may differ according to the social and psychological resources individuals have to draw upon, and the factors in their environments that may influence change in consumption. Generalized interventions targeting certain psychosocial risk factors may be unsuitable for individuals for whom these risks factors do not explain their behaviour. Tailored approaches could be most beneficial and better suited to those for whom MUP price intervention has not resulted in behaviour change.

### Study limitations

The key limitation of the study was the level of dropout and the potential for selective dropout. While the respondents were drinking at hazardous or harmful levels, consumption of ultra‐low‐price alcohol was not a daily occurrence for most participants. The respondents who provided too few data points for quantitative analysis or who dropped out from the study may have been drinking more heavily. Low response rates are a typical shortcoming of the EMA method [4], and thus we must consider that the patterns observed in this sample may be different among heavier drinkers. The findings should not be considered representative of response patterns at the population level. Identifying overall population changes are the focus of other MUP evaluation studies [2], and overall alcohol sales in Scotland have decreased since MUP [16]. The greater reduction in alcohol use among those drinking more heavily at baseline may be explained by regression to the mean. Supplementary analysis in the Supporting information Appendix S1 suggests that this may account for some, but not all, the observed between‐person variability in pre–post MUP change.

### Implications for research

In this study we have explored predictors of drinking and substance use looking at MUP influence on time‐specific data patterns. Future research could explore the potential of providing personalized interventions that address person‐specific predictors of drinking and substance use. For instance, for people who drink most when stressed, specific interventions should address not only drinking behaviour but also coping strategies for dealing with stressful situations. For people who are most prone to problematic drinking when in the presence of specific individuals, network intervention approaches may help in changing social connections to limit time spent in high‐risk social situations or modifying their social interactions to mitigate the risk in social encounters [17]. Implementing N‐of‐1 approaches to inform treatment, for example as part of community rehabilitation services could help with tailoring individual goals and relapse prevention strategies. This could be of particular importance among those for whom MUP has not reduced drinking.

N‐of‐1 is a recommended method for testing behavioural theory within individuals through repeated measures [18]. Our study showed how N‐of‐1 can be conducted in community settings with alcohol‐dependent groups. Behavioural sciences lack a long‐standing tradition of N‐of‐1 studies, and this design has been under‐used [19]. We have measured the predictors of drinking and substance use; however, we have not explored the consequences of drinking [20]. Future research could also look at individual‐level consequences, such as physiological states or social consequences such as violence or strained relationships.

One of the overarching questions for the MUP evaluation is ‘are some people and businesses more affected (positively or negatively) than others?’. The findings of our study suggest that subgroups of the population may be differentially affected by MUP implementation. Those who have fewer coping strategies may place themselves in debt or greater financial strain to obtain alcohol, while those with better coping strategies may utilize price, and improved health, as motivators to change drink type or to reduce the amount of alcohol consumed.

## Conclusions

The findings of this study provide greater insight into the variety of social and psychological processes which may contribute to changes in alcohol consumption at the individual level, and thus the change processes potentially underpinning population‐level change following MUP implementation. The study supports understanding the processes through which MUP may have its effect on population consumption, identifying possible mechanisms that lead to some heavy drinkers being more or less affected by the policy, and suggests avenues for further intervention approaches to reduce alcohol harm.

## Declaration of interests

None.

## Author contributions

**Dominika Kwasnicka:** Conceptualization; formal analysis; funding acquisition; investigation; methodology; project administration; supervision. **Massoud Boroujerdi:** Data curation; formal analysis; methodology; validation. **Aileen O'Gorman:** Formal analysis; funding acquisition; investigation; methodology. **Martin Anderson:** Data curation; formal analysis; methodology. **Peter Craig:** Data curation; funding acquisition; methodology. **Louise Bowman:** Data curation; investigation; project administration; supervision. **Mark McCann:** Conceptualization; data curation; formal analysis; funding acquisition; investigation; methodology; project administration; supervision; validation; visualization.

## Supporting information


**Data S1** Supporting Information.

## Footnotes

^1^ The Supporting information, Appendix S1 also reports on substance use and contacting treatment services, and a separate paper will focus on the importance of wider social context.
