# Supplementary material for: An N‐of‐1 study of daily alcohol consumption following minimum unit pricing implementation in Scotland
Source: Addiction. 2021 Jan 12;116(7):1725–33. doi: 10.1111/add.15382 (PMC8248017; doi:10.1111/add.15382)
Supplement: Supplementary file 1 — Data S1 Supporting Information. [file ADD-116-1725-s001.docx]

# An N-of-1 study of the psychosocial determinants of stopping, switching and seeking treatment behaviour following Minimum Unit Pricing implementation

# Appendix materials

###

### Appendix 1: Delphi method for developing survey questions

### Delphi advisors

We invited a range of experts to provide feedback on our questions; first, by e-mailing the initial question set, and second, by attending a Delphi workshop. We invited participants with a range of backgrounds and expertise, and the final set of advisors (N = 10) had backgrounds in: quantitative and qualitative alcohol research, substance treatment policy, policy evaluation research, addiction treatment, public health, N of 1 study design, Scottish Government policy, and peer research.

### Developing the initial question set

The research team developed a set of initial questions for inclusion in the study based on A.) a systematic review of the theoretical mechanisms underpinning behaviour change (Kwasnicka et al., 2016), B.) the theory of change for how MUP may affect alcohol and other drug use, and seeking treatment, and C.) interpersonal social factors affecting behaviour. From the behaviour change theory review, we developed questions around: maintenance motives; self-regulation; resources; habit; and environmental and social influence. From the MUP logic model, we included questions about alcohol and other drug use, use of services, and financial strain/displacement of spending. The process led to a set of 25 questions for consideration by Delphi participants and peer advisors.

###

### E-mail survey consultation

The initial set of questions was e-mailed to those invited to the delphi workshop. Some invitees and all workshop attendees provided feedback on the questions; and one respondent collated feedback from alcohol treatment service users and peer researchers. In the e-mail, we gave the following guidance:

- The questions are based on theories of **behaviour change maintenance,** **social support, and alcohol price.** Please tell us if we have missed something important.
- We are looking at things that could change a lot from one day to the next, so we can study change over 12 weeks.
- We will not ask things that do not change a lot over 12 weeks, even though they might be important (for example, living alone).
- We want the questions to be easy to understand, so people can answer the questions quickly every day.
- It is more important for us to find out about **change** in individual level of alcohol and drug use, rather than measuring how much people use.
- We want the survey to be as short as possible, so **we will remove some questions**, please tell us which questions we should not include.
- We will send a text asking participants to answer the questions around 7pm each day.
- The questions will appear in a random order each day.

For each question, we presented the survey item as it would appear in the online survey, along with three questions.

**Appendix Figure 1: Example question from the delphi process email consultation**


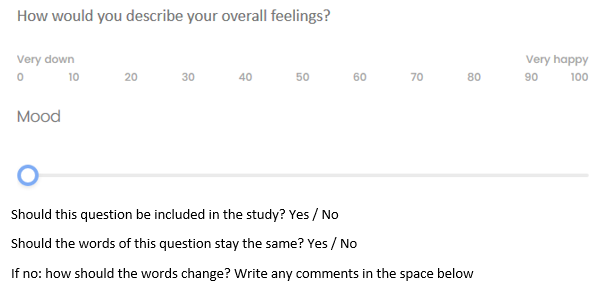


The responses from the survey were collated and then formed the discussion format for the Delphi workshop. If feedback was unanimous to retain a question unchanged, or to delete it, then the question would be retained or removed as appropriate. This did not occur, as all question had some variation or suggestion for a modification of the terms.

Based on the collated feedback, the wording of questions was changed, and additional questions added. A question around withdrawal symptoms were missed from the theoretical review but identified by service user representatives as important, and several rephrasing of original questions were made.

### Delphi workshop

At the Delphi workshop on the 11^th^ January 2018 (N=10), the participants were presented with 29 slides, with new wording for previous questions and additional questions. Participants then voted anonymously using radio voting buttons for the inclusion of each question, or for its change. After voting was completed, the results of the voting were counted, and the outcome discussed. Unanimous inclusions were retained in the final survey, and all other questions were discussed among the group. Peter Craig chaired the discussions while Mark McCann kept notes and proposed modified wording for questions and the addition of new questions. After discussion, a further round of voting took place, with unanimous decisions carried forward. There was one final round of discussions on the wording for remaining questions – all of which were unanimously agreed apart from one question. This question related to the degree to which individuals reported making efforts to control their drinking. The delphi group put forward five variations on the question and agreed that the final decision would be made based on a service user feedback (see ST1).

**Appendix Table 1: Example of the evolution of survey items through the Delphi process**

| Initial question | How much have you tried to control your drinking? |
| --- | --- |
| Delphi suggestions | How much have you been controlling your drinking? How much were you making an effort to reduce or stop drinking, or stay stopped? How much have you done to reduce or stop drinking, or stay stopped? How much have you used strategies to reduce or stop drinking, or stay stopped? How much have you tried to reduce or stop drinking, or stay stopped? |
| Final wording | How much have you tried to reduce or stop drinking, or stay stopped? |

The discussions at the Delphi workshop covered a range of topics, which are summarised below.

- **N of 1 question scales:** Information on categorical or binary issues were collected with a Yes/No answer followed up with a continuous scale question e.g. rating their intensity of drug use, or the positive/negative nature of interactions with people they had met
- **Understandability**: simplifying language where possible
- **Negative interpretations:** changing phrasing to avoid blaming, stigmatising or dramatizing language (e.g., around temptation, self-control)
- **Policy-neutral questions:** Framing questions that would a.) relate to MUP’s influence on alcohol affordability, b.) that would be applicable before and after MUP implementation, and c.) that weren’t “leading questions” that invited respondents to make an attribution of their behaviour to MUP.

**Appendix Table 2.** Questions included in the survey, the conceptual domain they relate to, and the format in which the questions were answered.

| Concept | Question | | | Answer format |
| --- | --- | --- | --- | --- |
| Resources | Over the last 24 hours, how would you rate your **mood**? | | | 0 - Very down, 100 – Very happy |
| Resources | Over the last 24 hours, how **stressed** did you feel? | | | 0 – Not at all, 100 – Very stressed |
| Resources | Have you experienced withdrawal? | | | Yes/No |
| Resources | How would you rate your withdrawal symptoms? | | | 0 – Very mild, 100 - Severe |
| Motives | How do you feel about your **drinking over the last 24 hours**? | | | 0 – Very negative, 100 – Very positive |
| Motives | How **motivated** did you feel to reduce or stop drinking, or stay stopped? | | | 0 - Not at all, 100 – Very |
| Regulation | How **tempted** were you to have a drink? | | | 0 - Not at all, 100 – Very |
| Motives | How much have you tried to **reduce or stop drinking, or stay stopped**? | | | 0 - Not at all, 100 – a lot |
| Environment | Over the last 24 hours, have you met up with? | | Friends; Family/Partners/Children; Professional Workers;  Support group peers; Others | |
| Environment | How did you get on with [Friends/ Family / Professionals / Support group / Others] over the last 24 hours? | | 0 - Very negative, 100 - Very positive | |
| Environment | How much did money influence **the amount you drank**? | | 0 – Not at all, 100 – A lot | |
| Environment | How much **did money influence the type** of alcohol you drank? | | 0 - Not at all, 100 – A lot | |
| Environment | How much of the time were you in situations where **alcohol was available** to you? | | 0 – Not at all, 100 – All the time | |
| Environment | How did money influence how much of the drug you used? | | 0 - Not at all, 100 - A lot | |
| Environment | How did money influence the type of drugs you used? | | 0 - Not at all, 100 - a lot | |
| Environment | Did you get any help or support from AA / mutual aid over the last 24 hours? | | 0 – Not at all, 100 – spent almost all day | |
| Outcome | Did you have an **alcoholic drink** in the last 24 hours? | Yes/No | | |
| Outcome | Where did you drink? | (At home; In pub/club/restaurant; Someone’s house; Outdoors; Other (Write in) | | |
| Outcome | Did you drink: | Beer, Cider, Spirits, Wine  Other | | |
| Outcome | How much did you drink? | If drank in pub/club restaurant  Pints beer; Pints cider; Measures Spirits; Glasses Wine  If drank in other places  Cans Beer; Litres Cider; (Half) bottles spirits; Bottles Wine; Other | | |
| Outcome | Did you **take drugs** or other mood-altering substances in the last 23 hours? | Yes / No | | |
| Outcome | If yes, did you take: | Cannabis; Cocaine; Ecstasy; Solvents / Gases; Valium / Diazepam / other downers; Speed; Heroin; Methadone; Suboxone/Subutex; Pregabalin/Gabapentin; Other [Write in] | | |
| Outcome | How would you describe your use of *drug name* over the last 24 hours? | 0 – A bit, 100 – A lot | | |
| Outcome | Did you get any help or support from drug & alcohol services? | 0 – Not at all, 100 – spent almost all day | | |
| Outcome | Did you seek help from drug & alcohol services in the last 24 hours? | Yes /No | | |
| Open response format | Please tell us about anything that affected how you were feeling, or that had an impact on your drinking that we haven’t asked about | Open text – question always appeared as last and was not randomised | | |

*Note.* The answer format for the drink questions were “half-bottle”, and then whole numbers up until “ten or more”. This mean that, for respondents who drank more than ten cans of beer in a day, the calculation of their total alcohol consumption was an underestimate of the true amount.

### Appendix 2: Visual plots of survey responses

The diagrams below present changes in respondents’ responses to selected study variables over time. The variables presented below are the ones that were answered most fully by respondents; some respondents did not report alcohol or drug use, hence there were no responses for these individuals. Figure 10 shows the mood scores for respondents recruited in Wave 1. It is also clear that there are large between-person differences in the overall rating of mood, and its variability from day to day. 1002 and 1005 consented to continue the study into the following waves, while the other respondents had varying lengths of participation. The following figures show scores for mood, stress, motivation, temptations and effort.

**Appendix Figure 2: Wave 1 Mood scores over calendar time**

**Appendix Figure 3: Wave 2 Mood scores over calendar time**

**Appendix Figure 4: Wave 3 Mood scores over calendar time**

**Appendix Figure 5: Wave 1: Stress scores over calendar time**

**Appendix Figure 6: Wave 2 stress scores over calendar time**

**Appendix Figure 7: Wave 3 Stress scores over calendar time**

**Appendix Figure 8: Wave 1 Motivation scores over calendar time**

**Appendix Figure 9: Wave 2 Motivation scores over calendar time**

**Appendix Figure 10: Wave 3: Motivation scores over calendar time**

**Appendix Figure 11: Wave 1 Tempted scores over calendar time**

**Appendix Figure 12: Wave 2 Tempted scores over calendar time**

**Appendix Figure 13: Wave 3: Tempted scores over calendar time**


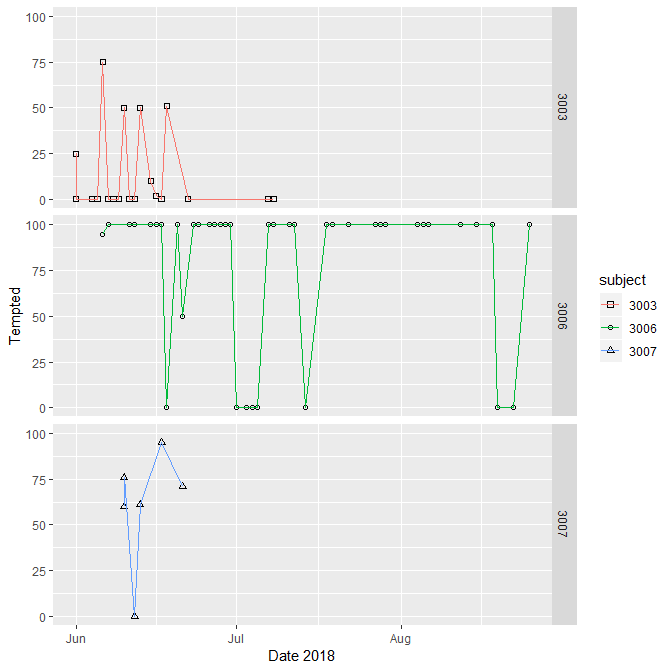


**Appendix Figure 14: Wave 1 Effort scores over calendar time**

**Appendix Figure 15: Wave 2 Effort scores over calendar time**

**Appendix Figure 16: Wave 3: Effort scores over calendar time**

**Appendix 3: Descriptive statistics and additional analyses**

**Appendix Table 3.** Characteristics of respondents at baseline recruitment

| **ID** | **Gender** | **Age** | **Other substances** | **Stopped or controlling**  **alcohol use** | **Employment status** | **Accommodation status (Independent, supported, homeless)** | Living alone | Recent social contact | Social Outcomes Index |
| --- | --- | --- | --- | --- | --- | --- | --- | --- | --- |
| 1002 | Male | 46-50 | Co-codamol | Yes | Not working | Independent | Yes | No | 3 |
| 1003 | Male | 46-50 |  |  | Employed | Independent |  | Yes | 5 |
| 1004 | Female | 55-60 |  | Yes | Employed | Independent |  |  | 4 |
| 1005 | Female | 50-55 | Cannabis |  | Not working | Independent | Yes | Yes | 4 |
| 1006 | Male | 36-40 |  | Yes | Employed | Independent | Yes | Yes | 6 |
| 1007 | Female | 40-45 | Cannabis | Yes | Not working | Independent | Yes | Yes | 4 |
| 1008 | Female | 55-59 | Alcohol | Yes | Not working | Independent | Yes |  | 3 |
| 1009* | Other | 50-55 | Alcohol | Yes | Not working | Independent | Yes | Yes | 4 |
| 1011 | Male | 40-45 | Alcohol | Yes | Not working | Independent | Yes | Yes | 4 |
| 1012 | Female | 46-50 | Alcohol | Yes | Volunteer | Independent | Yes |  | 4 |
| 1013 | Male | 50-55 | Alcohol | Yes | Volunteer | Independent | Yes | Yes | 5 |
| 2001 | Male | 56-60 |  |  | Not working | Homeless | Yes | Yes | 2 |
| 2003* | Female | 60-65 | Alcohol | Yes | Not working | Independent |  | Yes | 3 |
| 2004* | Male | 66-70 |  |  | Not working | Independent |  | Yes | 3 |
| 2005 | Male | 36-40 |  | Yes | Employed | Independent | Yes | Yes | 6 |
| 2006 | Male | 50-55 |  | Yes | Employed | Independent | Yes | Yes | 6 |
| 2007* | Male | 60-65 |  | Yes | Not working | Independent | Yes | Yes | 4 |
| 2008 | Female | 40-45 | Valium |  | Not working | Independent |  | Yes | 3 |
| 2009 | Male | 36-40 | Heroin |  | Not working | Independent |  |  | 2 |
| 2010 | Male | 25-30 |  |  | Not working | Homeless | Yes | Yes | 2 |
| 2011* | Male | 60-65 | Dihydrocodeine | Yes | Not working | Independent | Yes | Yes | 4 |
| 2017 | Female | 40-45 |  | Yes | Not working | Independent | Yes | Yes | 4 |
| 3003 | Male | 30-35 |  | Yes | Not working | Independent | Yes | Yes | 4 |
| 3006 | Male | 30-35 |  |  | Not working | Independent |  | Yes | 3 |
| 3007* | Male | 16-20 |  |  | Not working | Independent |  | Yes | 3 |

* - These participants also provided qualitative follow up interview data

**Appendix Table 4:** Number of responses and descriptive statistics for daily survey measures - outcome measures

| **ID** | **N** | **Alcohol units**  **Mean^** | **Alcohol units**  **SD** | **Alcohol units**  **Median** | **Alcohol units**  **%missing** | **Taking drugs**  **% missing** | **% days**  **taking drugs** | **Seeking help**  **%missing** | **% of times**  **Seeking help** | **Drinking %missing** | **% of days**  **drinking** |
| --- | --- | --- | --- | --- | --- | --- | --- | --- | --- | --- | --- |
| 1002* | 109 | 1 | 7 | 0 | 0 | 0 | 99 | 11 | 33 | 0 | 1 |
| 1003 | 4 | 8 | 10 | 6 | 0 | 0 | 25 | 25 | 0 | 0 | 50 |
| 1004* | 45 | 0 | 3 | 0 | 0 | 2 | 0 | 9 | 29 | 0 | 2 |
| 1005* | 36 | 2 | 6 | 0 | 0 | 3 | 100 | 17 | 13 | 0 | 28 |
| 1006* | 22 | 7 | 10 | 0 | 0 | 0 | 0 | 5 | 0 | 0 | 36 |
| 1007* | 21 | 5 | 6 | 0 | 0 | 0 | 33 | 0 | 10 | 0 | 38 |
| 1008 | 2 | 5 | 7 | 5 | 0 | 0 | 0 | 0 | 0 | 0 | 50 |
| 1009* | 25 | 18 | 14 | 19 | 0 | 4 | 8 | 16 | 19 | 0 | 68 |
| 1011 | 3 | 8 | 14 | 0 | 0 | 0 | 100 | 33 | 0 | 0 | 33 |
| 1012* | 39 | 22 | 8 | 20 | 0 | 0 | 0 | 3 | 18 | 0 | 100 |
| 1013 | 8 | 42 | 22 | 36 | 0 | 0 | 12 | 12 | 29 | 0 | 100 |
| 2001* | 58 | 5 | 10 | 0 | 0 | 0 | 0 | 5 | 0 | 0 | 22 |
| 2003 | 3 | 6 | 6 | 10 | 0 | 33 | 50 | 33 | 0 | 0 | 67 |
| 2004* | 43 | 14 | 13 | 15 | 0 | 2 | 0 | 16 | 0 | 0 | 70 |
| 2005* | 33 | 14 | 10 | 20 | 0 | 0 | 48 | 3 | 0 | 0 | 70 |
| 2006* | 80 | 0 | 3 | 0 | 0 | 1 | 0 | 18 | 24 | 0 | 2 |
| 2007 | 7 | 5 | 6 | 0 | 0 | 14 | 0 | 14 | 33 | 0 | 43 |
| 2008 | 4 | 0 | 0 | 0 | 0 | 0 | 25 | 0 | 0 | 0 | 0 |
| 2009 | 6 | 2 | 4 | 0 | 0 | 0 | 0 | 17 | 0 | 0 | 17 |
| 2010 | 6 | 12 | 18 | 0 | 0 | 0 | 0 | 17 | 0 | 0 | 33 |
| 2011* | 15 | 23 | 7 | 24 | 0 | 0 | 80 | 33 | 50 | 0 | 100 |
| 2017* | 28 | 10 | 10 | 10 | 0 | 4 | 0 | 7 | 8 | 0 | 89 |
| 3003* | 21 | 3 | 8 | 0 | 0 | 5 | 85 | 19 | 18 | 0 | 14 |
| 3006* | 43 | 8 | 10 | 0 | 0 | 0 | 19 | 12 | 0 | 0 | 37 |
| 3007 | 7 | 45 | 53 | 15 | 0 | 0 | 57 | 14 | 0 | 0 | 71 |
| Note | *Note. ^*Mean units per drinking day. *We imputed data for these 15 respondents. | | | | | | |  |  |  |  |

Appendix Table 5 shows the model results relating to manuscript Figure 1. This model was run on the first imputed dataset of the 50 used in the main analysis. There was strong evidence to suggest adding the random slope parameter to the model was a better fit to the data than the random intercepts model (Likelihood ratio test Chi Square (2 d.f.) = 29.74; p < 0.0001).

**Appendix Table 5:** Random intercept - random slopes model for MUP on daily alcohol units

| **Parameter** | **Coefficients** |
| --- | --- |
| **Time** | -0.02 (-0.03, 0.00) |
| **Post-MUP** | -1.65 (-3.94, 0.64) |
| **Intercept** | 6.21 (3.65, 8.76) |
| **Random effects** |  |
| **Intercept variance** | 21.74 |
| **Slope variance** | 11.20 |
| **Intercept-Slope covariance** | -14.86 |

To assess the extent to which a regression to the mean effect may explain the relationship between higher initial alcohol use and decrease after MUP, we created a comparison analysis using pre-post the middle observation point for each participant, rather than pre-post MUP policy. This parameter effectively asks “was alcohol consumption different at the start compared to the end of the study for each participant” rather than “was alcohol consumption different for participants before and after MUP”. Appendix table 3 shows the results of this model. This model was run on the first imputed dataset of the 50 used in the main analysis. There was evidence to suggest that the random slope model was a better fit to the data than the random intercepts model (Likelihood ratio test Chi Square (2 d.f.) = 13.61; p = 0.001). The between-participant variation was much lower for the midpoint parameter than for the MUP parameter.

**Appendix Table 6:** Random intercept - random slopes model for middle observation point on daily alcohol units

| **Parameter** | **Coefficients** |
| --- | --- |
| **Time** | -0.03 (-0.05, -0.01) |
| **Post-midpoint** | -0.14 (-1.92, 1.64) |
| **Intercept** | 5.98 (3.71, 8.25) |
| **Random effects** |  |
| **Intercept variance** | 17.75 |
| **Slope variance** | 5.00 |
| **Intercept-Slope covariance** | -7.15 |

**Appendix Figure 17:** Individual differences in pre-post survey midpoint alcohol consumption for 15 participants who provided sufficient data for quantitative analysis

**Appendix Table 7:** Multilevel logistic regression model with drug use as dependent variable

| **Parameter** | **Model 1** | **Model 2** | **Model 3** | **Model 4** | **Model 5** | **Model 6** | **Model 7** | **Model 8** |
| --- | --- | --- | --- | --- | --- | --- | --- | --- |
| **Time** | -0.02 (-0.03, 0.00) | -0.02 (-0.03, 0.00) | -0.02 (-0.03, 0.00) | -0.02 (-0.03, 0.00) | -0.02 (-0.03, 0.00) | -0.02 (-0.03, 0.00) | -0.02 (-0.03, 0.00) | -0.02 (-0.03, 0.00) |
| **Post-MUP** | 0.72 (-0.38, 1.82) | 0.83 (-0.31, 1.97) | 0.71 (-0.39, 1.81) | 0.74 (-0.36, 1.84) | 0.72 (-0.38, 1.82) | 0.74 (-0.38, 1.85) | 0.72 (-0.38, 1.82) | 0.82 (-0.33, 1.97) |
| **Mood** |  | 0.01 (0.00, 0.02) |  |  |  |  |  | 0.01 (0.00, 0.03) |
| **Motivation** |  |  | 0.00 (-0.01, 0.01) |  |  |  |  | 0.00 (-0.02, 0.01) |
| **Tempted** |  |  |  | 0.00 (-0.01, 0.01) |  |  |  | 0.00 (-0.01, 0.01) |
| **Effort** |  |  |  |  | 0.00 (-0.01, 0.01) |  |  | 0.00 (-0.01, 0.01) |
| **Stress** |  |  |  |  |  | 0.00 (-0.02, 0.01) |  | 0.00 (-0.01, 0.02) |
| **Alcohol situations** |  |  |  |  |  |  | 0 (-0.01, 0.01) | 0.00 (-0.01, 0.01) |
| **Intercept** | -1.75 (-3.88, 0.38) | -2.24 (-4.47, -0.01) | -1.63 (-3.86, 0.61) | -1.58 (-3.81, 0.64) | -1.68 (-3.88, 0.52) | -1.62 (-3.90, 0.66) | -1.77 (-3.94, 0.40) | -2.25 (-5.13, 0.63) |
| **Level 2 variance** | 14.41 | 14.58 | 14.62 | 14.3 | 14.54 | 14.54 | 14.51 | 15.00 |
| **ICC** | 0.81 | 0.82 | 0.82 | 0.81 | 0.82 | 0.82 | 0.82 | 0.82 |

The confidence intervals overlapping the null for post-MUP in Table 3 suggest that there is no evidence that this participant group, collectively, changed the number of days they used drugs before and after MUP implementation.

**Appendix Table 8:** Multilevel logistic regression model with seeking help as dependent variable

| **Parameter** | **Model 1** | **Model 2** | **Model 3** | **Model 4** | **Model 5** | **Model 6** | **Model 7** | **Model 8** |
| --- | --- | --- | --- | --- | --- | --- | --- | --- |
| **Time** | 0 (-0.01, 0.01) | 0 (-0.01, 0.01) | 0 (-0.01, 0.01) | 0 (-0.01, 0.01) | 0 (-0.01, 0.01) | 0 (-0.01, 0.01) | 0 (-0.01, 0.01) | 0 (-0.01, 0.01) |
| **Post-MUP** | -0.51 (-1.34, 0.33) | -0.5 (-1.34, 0.34) | -0.52 (-1.35, 0.31) | -0.47 (-1.31, 0.37) | -0.51 (-1.35, 0.33) | -0.51 (-1.34, 0.33) | -0.53 (-1.36, 0.29) | -0.49 (-1.33, 0.35) |
| **Mood** |  | 0.01 (0, 0.02) |  |  |  |  |  | 0.01 (-0.01, 0.02) |
| **Motivation** |  |  | 0.01 (0, 0.02) |  |  |  |  | 0 (-0.01, 0.01) |
| **Tempted** |  |  |  | -0.01 (-0.02, 0) |  |  |  | -0.01 (-0.01, 0) |
| **Effort** |  |  |  |  | 0.01 (0, 0.02) |  |  | 0.01 (-0.01, 0.02) |
| **Stress** |  |  |  |  |  | 0 (-0.01, 0.01) |  | 0.01 (0, 0.02) |
| **Alcohol situations** |  |  |  |  |  |  | 0 (-0.01, 0.00) | 0 (-0.01, 0.00) |
| **Intercept** | -2.36 (-3.4, -1.33) | -2.74 (-3.91, -1.56) | -2.76 (-3.93, -1.59) | -1.94 (-3.05, -0.82) | -3.00 (-4.29, -1.71) | -2.38 (-3.52, -1.24) | -2.18 (-3.19, -1.17) | -3.11 (-3.11, -3.11) |
| **Level 2 variance** | 2.76 | 2.85 | 2.51 | 2.65 | 2.37 | 2.76 | 2.35 | 2.18 |
| **ICC** | 0.46 | 0.46 | 0.43 | 0.45 | 0.42 | 0.46 | 0.42 | 0.40 |

Table 8 shows the model results with daily seeking support from services as the outcome. The confidence intervals crossing the null suggest there is little evidence of change in support seeking before and after MUP for this participant group. None of the other variables were predictive of help seeking

**Appendix Table 9:** Number of responses and descriptive statistics for daily survey measures part 1.

| ID | Response days | Tempted  mean | Tempted SD | Tempted median | Tempted  %missing | Stress  mean | Stress  SD | Stress  median | Stress  %missing | Effort  mean | Effort  SD | Effort median | Effort %missing |
| --- | --- | --- | --- | --- | --- | --- | --- | --- | --- | --- | --- | --- | --- |
| 1002 | 109 | 21 | 28 | 11 | 10 | 61 | 22 | 67 | 17 | 100 | 3 | 100 | 10 |
| 1003 | 4 | 30 | 26 | 39 | 25 | 59 | 45 | 68 | 0 | 26 | 34 | 26 | 50 |
| 1004 | 45 | 33 | 27 | 26 | 18 | 53 | 25 | 50 | 16 | 98 | 12 | 100 | 16 |
| 1005 | 36 | 45 | 34 | 50 | 17 | 59 | 28 | 67 | 17 | 91 | 22 | 100 | 19 |
| 1006 | 22 | 48 | 25 | 61 | 23 | 54 | 16 | 60 | 9 | 54 | 28 | 62 | 27 |
| 1007 | 21 | 55 | 42 | 80 | 19 | 70 | 29 | 84 | 5 | 59 | 36 | 74 | 14 |
| 1008 | 2 | 20 | 6 | 20 | 0 | 16 | 19 | 16 | 0 | 0 | NA | 0 | 50 |
| 1009 | 25 | 82 | 27 | 96 | 4 | 75 | 21 | 80 | 20 | 26 | 37 | 4 | 32 |
| 1011 | 3 | 51 | 42 | 36 | 0 | 30 | 37 | 18 | 0 | 100 | 0 | 100 | 33 |
| 1012 | 39 | 87 | 17 | 90 | 15 | 88 | 14 | 88 | 5 | 19 | 25 | 14 | 13 |
| 1013 | 8 | 62 | 35 | 78 | 38 | 48 | 37 | 38 | 12 | 7 | 10 | 0 | 0 |
| 2001 | 58 | 66 | 8 | 65 | 22 | 64 | 7 | 64 | 7 | 54 | 12 | 57 | 10 |
| 2003 | 3 | 48 | NA | 48 | 67 | 50 | 71 | 50 | 33 | 45 | 64 | 45 | 33 |
| 2004 | 43 | 67 | 20 | 70 | 21 | 60 | 18 | 63 | 14 | 54 | 20 | 55 | 5 |
| 2005 | 33 | 90 | 18 | 100 | 9 | 68 | 23 | 75 | 21 | 59 | 44 | 75 | 9 |
| 2006 | 80 | 40 | 31 | 32 | 8 | 45 | 25 | 50 | 9 | 96 | 14 | 100 | 15 |
| 2007 | 7 | 58 | 45 | 70 | 14 | 35 | 41 | 20 | 29 | 93 | 12 | 100 | 14 |
| 2008 | 4 | 50 | 58 | 50 | 0 | 0 | 0 | 0 | 25 | 33 | 58 | 0 | 25 |
| 2009 | 6 | 30 | 45 | 0 | 17 | 17 | 41 | 0 | 0 | 80 | 45 | 100 | 17 |
| 2010 | 6 | 70 | 41 | 75 | 17 | 31 | 38 | 15 | 0 | 0 | 0 | 0 | 33 |
| 2011 | 15 | 99 | 1 | 100 | 7 | 82 | 16 | 86 | 0 | 92 | 8 | 91 | 13 |
| 2017 | 28 | 87 | 20 | 94 | 14 | 44 | 37 | 30 | 14 | 22 | 34 | 6 | 18 |
| 3003 | 21 | 15 | 24 | 0 | 14 | 48 | 34 | 50 | 10 | 85 | 22 | 100 | 10 |
| 3006 | 43 | 79 | 40 | 100 | 5 | 70 | 25 | 75 | 9 | 70 | 45 | 100 | 9 |
| 3007 | 7 | 57 | 35 | 61 | 29 | 13 | 32 | 0 | 14 | 24 | 36 | 12 | 14 |

**Appendix Table 10:** Number of responses and descriptive statistics for daily survey measures part 2.

| ID | Mood  mean | Mood  SD | Mood median | Mood  %  Missing | Motivated  mean | Motivated  SD | Motivated median | Motivated  %missing | Drink  feeling mean | Drink  feeling SD | Drink  feeling median | Drink  feeling % missing |
| --- | --- | --- | --- | --- | --- | --- | --- | --- | --- | --- | --- | --- |
| 1002 | 67 | 22 | 73 | 8 | 87 | 24 | 100 | 12 | 50 | NA | 50 | 99 |
| 1003 | 38 | 31 | 38 | 0 | 58 | 42 | 64 | 0 | 74 | 34 | 74 | 50 |
| 1004 | 65 | 19 | 74 | 16 | 86 | 24 | 100 | 9 | 2 | NA | 2 | 98 |
| 1005 | 59 | 25 | 51 | 6 | 93 | 14 | 100 | 6 | 58 | 33 | 50 | 75 |
| 1006 | 58 | 13 | 63 | 5 | 61 | 21 | 65 | 14 | 45 | 20 | 44 | 64 |
| 1007 | 34 | 35 | 15 | 10 | 47 | 35 | 53 | 14 | 13 | 8 | 14 | 62 |
| 1008 | 23 | NA | 23 | 50 | 49 | 69 | 49 | 0 | 50 | NA | 50 | 50 |
| 1009 | 37 | 24 | 39 | 8 | 28 | 34 | 17 | 16 | 44 | 23 | 50 | 32 |
| 1011 | 63 | 9 | 60 | 0 | 74 | 32 | 84 | 0 | 10 | NA | 10 | 67 |
| 1012 | 7 | 9 | 2 | 8 | 30 | 35 | 18 | 28 | 10 | 20 | 0 | 0 |
| 1013 | 36 | 29 | 24 | 0 | 20 | 29 | 0 | 12 | 19 | 14 | 20 | 0 |
| 2001 | 46 | 13 | 45 | 17 | 47 | 11 | 46 | 10 | 45 | 9 | 46 | 78 |
| 2003 | 42 | NA | 42 | 67 | 24 | 35 | 24 | 33 | 32 | 26 | 32 | 33 |
| 2004 | 47 | 17 | 50 | 14 | 54 | 19 | 50 | 12 | 40 | 12 | 42 | 30 |
| 2005 | 50 | 22 | 50 | 9 | 64 | 37 | 71 | 15 | 43 | 29 | 50 | 30 |
| 2006 | 54 | 25 | 50 | 14 | 96 | 12 | 100 | 15 | 2 | 3 | 2 | 98 |
| 2007 | 53 | 35 | 56 | 43 | 68 | 41 | 70 | 29 | 40 | 17 | 50 | 57 |
| 2008 | 0 | 0 | 0 | 25 | 0 | 0 | 0 | 25 | NA | NA | NA | 100 |
| 2009 | 70 | 45 | 100 | 17 | 75 | 42 | 100 | 0 | 34 | NA | 34 | 83 |
| 2010 | 0 | 0 | 0 | 33 | 14 | 12 | 18 | 0 | 80 | 6 | 80 | 67 |
| 2011 | 30 | 20 | 24 | 7 | 86 | 27 | 94 | 7 | 19 | 25 | 15 | 0 |
| 2017 | 64 | 29 | 71 | 4 | 36 | 37 | 18 | 14 | 57 | 23 | 67 | 11 |
| 3003 | 49 | 32 | 50 | 14 | 87 | 18 | 100 | 29 | 51 | 19 | 50 | 86 |
| 3006 | 37 | 26 | 50 | 16 | 81 | 35 | 100 | 7 | 30 | 30 | 36 | 63 |
| 3007 | 78 | 35 | 100 | 14 | 41 | 35 | 50 | 0 | 53 | 35 | 50 | 29 |

**Appendix Table 11**: Number of responses and descriptive statistics for daily survey measures part 3.

| ID | Alcohol  units mean | Alcohol  units  SD | Alcohol  units median | Alcohol units  %missing | Situations mean | Situations SD | Situations  median | Situations  %missing | Period  (days) |
| --- | --- | --- | --- | --- | --- | --- | --- | --- | --- |
| 1002 | 1 | 7 | 0 | 0 | 3 | 4 | 1 | 8 | 146 |
| 1003 | 8 | 10 | 6 | 0 | 50 | 41 | 50 | 0 | 8 |
| 1004 | 0 | 3 | 0 | 0 | 42 | 22 | 50 | 18 | 77 |
| 1005 | 2 | 6 | 0 | 0 | 32 | 38 | 0 | 8 | 149 |
| 1006 | 7 | 10 | 0 | 0 | 69 | 4 | 68 | 9 | 50 |
| 1007 | 5 | 6 | 0 | 0 | 13 | 27 | 2 | 19 | 70 |
| 1008 | 5 | 7 | 5 | 0 | 4 | 5 | 4 | 0 | 1 |
| 1009 | 18 | 14 | 19 | 0 | 73 | 35 | 90 | 12 | 48 |
| 1011 | 8 | 14 | 0 | 0 | 80 | 28 | 80 | 33 | 4 |
| 1012 | 22 | 8 | 20 | 0 | 95 | 8 | 100 | 15 | 86 |
| 1013 | 42 | 22 | 36 | 0 | 86 | 11 | 84 | 12 | 59 |
| 2001 | 5 | 10 | 0 | 0 | 74 | 9 | 75 | 16 | 81 |
| 2003 | 6 | 6 | 10 | 0 | 60 | 57 | 60 | 33 | 59 |
| 2004 | 14 | 13 | 15 | 0 | 82 | 15 | 79 | 5 | 86 |
| 2005 | 14 | 10 | 20 | 0 | 51 | 27 | 50 | 15 | 47 |
| 2006 | 0 | 3 | 0 | 0 | 12 | 17 | 7 | 15 | 125 |
| 2007 | 5 | 6 | 0 | 0 | 69 | 40 | 86 | 29 | 69 |
| 2008 | 0 | 0 | 0 | 0 | 12 | 25 | 0 | 0 | 9 |
| 2009 | 2 | 4 | 0 | 0 | 12 | 22 | 0 | 17 | 31 |
| 2010 | 12 | 18 | 0 | 0 | 31 | 38 | 14 | 0 | 13 |
| 2011 | 23 | 7 | 24 | 0 | 94 | 18 | 100 | 7 | 134 |
| 2017 | 10 | 10 | 10 | 0 | 97 | 7 | 100 | 25 | 42 |
| 3003 | 3 | 8 | 0 | 0 | 8 | 16 | 0 | 10 | 54 |
| 3006 | 8 | 10 | 0 | 0 | 41 | 19 | 50 | 19 | 82 |
| 3007 | 45 | 53 | 15 | 0 | 72 | 26 | 75 | 0 | 63 |

**Appendix Table 12:** Proportion of outcomes more than one standard deviation from the mean.

| ID | Tempted | Stress | Effort | Mood | Motivation | Situations | Drink Feeling | Alcohol units |
| --- | --- | --- | --- | --- | --- | --- | --- | --- |
| 1002 | 0.17 | 0.2 | 0.02 | 0.23 | 0.1 | 0.1 | 0 | 0.01 |
| 1003 | 0.25 | 0.25 | 0 | 0.5 | 0.25 | 0.5 | 0 | 0.25 |
| 1004 | 0.24 | 0.24 | 0.02 | 0.18 | 0.16 | 0.2 | 0 | 0.02 |
| 1005 | 0.31 | 0.22 | 0.08 | 0.28 | 0.11 | 0.22 | 0.06 | 0.14 |
| 1006 | 0.18 | 0.23 | 0.18 | 0.27 | 0.18 | 0.36 | 0.14 | 0.32 |
| 1007 | 0.24 | 0.19 | 0.29 | 0.24 | 0.38 | 0.1 | 0.19 | 0.38 |
| 1008 | 0 | 0 | 0 | 0 | 0 | 0 | 0 | 0 |
| 1009 | 0.16 | 0.48 | 0.12 | 0.28 | 0.16 | 0.12 | 0.16 | 0.44 |
| 1011 | 0.33 | 0.33 | 0 | 0.33 | 0.33 | 0 | 0 | 0.33 |
| 1012 | 0.1 | 0.05 | 0.1 | 0.13 | 0.18 | 0.1 | 0.08 | 0.33 |
| 1013 | 0.12 | 0.38 | 0.25 | 0.38 | 0.12 | 0.5 | 0.38 | 0.25 |
| 2001 | 0.16 | 0.21 | 0.33 | 0.28 | 0.21 | 0.19 | 0.09 | 0.14 |
| 2003 | 0 | 0 | 0 | 0 | 0 | 0 | 0 | 0 |
| 2004 | 0.16 | 0.12 | 0.26 | 0.16 | 0.3 | 0.35 | 0.09 | 0.44 |
| 2005 | 0.12 | 0.21 | 0.27 | 0.39 | 0.18 | 0.21 | 0.24 | 0.33 |
| 2006 | 0.34 | 0.35 | 0.04 | 0.34 | 0.05 | 0.08 | 0 | 0.02 |
| 2007 | 0.29 | 0.14 | 0.14 | 0.29 | 0.14 | 0.14 | 0.14 | 0.43 |
| 2008 | 0 | 0 | 0.25 | 0 | 0 | 0.25 | 0 | 0 |
| 2009 | 0.17 | 0.17 | 0.17 | 0.17 | 0.17 | 0.17 | 0 | 0.17 |
| 2010 | 0.17 | 0.33 | 0 | 0 | 0.33 | 0.33 | 0 | 0.17 |
| 2011 | 0.07 | 0.13 | 0.13 | 0.2 | 0.13 | 0.07 | 0.07 | 0.2 |
| 2017 | 0.07 | 0.43 | 0.18 | 0.29 | 0.21 | 0.07 | 0.29 | 0.21 |
| 3003 | 0.19 | 0.33 | 0.24 | 0.29 | 0.1 | 0.14 | 0 | 0.1 |
| 3006 | 0.19 | 0.33 | 0.26 | 0.35 | 0.12 | 0.14 | 0.02 | 0.37 |
| 3007 | 0.29 | 0.14 | 0.14 | 0.14 | 0.43 | 0.43 | 0.29 | 0.29 |

**Appendix 4: Further analysis of factors related to consumption change after MUP**

**Appendix Table 13:** Regression coefficients showing the standard deviation change in daily units after MUP, and the change in the post-MUP coefficient after accounting for psychosocial factors. Four participants with least change in consumption after MUP

|  | 1002 | | | 1005 | | | 2006 | | | 2011 |  |  |
| --- | --- | --- | --- | --- | --- | --- | --- | --- | --- | --- | --- | --- |
|  | Coef | P value | Coef change | Coef | P value | Coef change | Coef | P value | Coef change | Coef | P value | Coef change |
| Model |  |  |  |  |  |  |  |  |  |  |  |  |
| Unadjusted | -0.12 | 0.454 | 0 | 0.169 | 0.296 | 0 | 0.199 | 0.329 | 0 | 0.199 | 0.442 | 0 |
| + Mood | ~~~ | ~~~ | ~~~ | 0.141 | 0.405 | 0.17 | 0.185 | 0.355 | 0.07 | ~~~ | ~~~ | ~~~ |
| + Motivation | -0.02 | 0.897 | 0.83 | -0.095 | 0.576 | 1.56 | 0.176 | 0.31 | 0.12 | ~~~ | ~~~ | ~~~ |
| + Tempted | 0.001 | 0.993 | 1.01 | -0.131 | 0.454 | 1.78 | 0.166 | 0.337 | 0.17 | ~~~ | ~~~ | ~~~ |
| + Effort | 0 | 0.996 | 1 | -0.176 | 0.335 | 2.04 | -0.14 | 0.185 | 1.7 | ~~~ | ~~~ | ~~~ |
| + Stress | ~~~ | ~~~ | ~~~ | -0.167 | 0.361 | 1.99 | -0.14 | 0.185 | 1.7 | ~~~ | ~~~ | ~~~ |
| + Alcohol situations | ~~ | ~~~ | ~~~ | 0.007 | 0.966 | 0.96 | -0.14 | 0.185 | 1.7 | -0.103 | 0.411 | 1.52 |
